# Announcement

**Published:** 1886-10

**Authors:** 


### EDITORIAL DEPARTMENT.

### ANNOUNCEMENT.

Our readers will notice that with this number, the journal presents several important changes. The business management has been transferred to Philadelphia, and the senior editor has associated with him Dr. Huidekoper, of the Veterinary Department of the University of Pennsylvania. A systematic effort has been made to secure the co-operation of a number of well-known men, who, participating actively in the work of the JOURNAL, will represent the scientific as well as the practical aspects of comparative medicine, in the broadest sense of this term. We point with pleasure to the announcement of the staff of collaborators, on which will be found the names of the most representative men in the country in their various departments. The association of medical men with this undertaking has always been very close; it originated, indeed, in the zeal and ambition of a physician, Dr. Spitzka, who felt that the workers in comparative medicine in this country should have an organ worthy to be compared with the European journals. This continued interest of members of the medical profession is assured by the names of the distinguished men which appear on our title page.

The scope and objects of the JOURNAL will continue to be the same.

It will offer a medium for the publication of important original communications. The seven years which have elapsed since the foundation of the JOURNAL have been important ones in the history of American Veterinary Medicine. New schools have been founded, the old ones have been strengthened, and there is everywhere so much more activity displayed in the investigation of disease, that we confidently anticipate an increasing number of valuable memoirs from the laboratories and clinics of American schools. To our contributors in this department, we can promise prompt publication and a wide circulation of their articles among an interested class of readers. We shall also, from time to time, as in the present number, publish valuable communications from foreign writers. A clinical department will present summaries of interesting cases, notes on treatment and abstracts of post-mortems. We have arranged with several of the leading clinics of the country, to furnish such reports, and our columns will always be open for the reception, from any source, of short, pithy, practical notes on treatment.

In the Review department we shall give careful criticisms of recent native and foreign works, and from time to time summaries of progress in the branches of pathology, surgery and anatomy.

The editorial columns will deal with questions of scientific and general interest, as they arise, treated from an independent stand-point; and we shall make the advancement of the study and practice of comparative medicine the first consideration, to which all others shall be subsidiary. Our highest ambition is to continue the JOURNAL as the scientific organ of the veterinary profession, and we hopefully anticipate the support of all who have its interests at heart.

A bright future we believe lies before the veterinary science and art in this country. Conditions and contingencies, beyond the control of the wisest, have thwarted and hampered those who, knowing what was highest, chafed at professional life and work so far below their ideal standards; but the old order changes, and we look with pride to the rapid growth of the profession, to the attainment of a general excellence among practitioners, commensurate with the importance of their duties and to the development of a special excellence among the scientific workers, which shall give to this country a leading position in all that relates to comparative medicine.

